# Effects of transport stress on pathological injury and expression of main heat shock proteins in the caprine stomach

**DOI:** 10.1186/s12917-020-02569-z

**Published:** 2020-09-22

**Authors:** Wei Hu, Tian Ye, Yanzhen Yang, Ben Liu, Wenya Zheng

**Affiliations:** 1 College of Life Science and Resources and Environment, Yichun University, Yichun, 336000 Jiangxi China; 2 Jiangxi Lvke Agriculture and Animal Husbandry Technology co. LTD, Yichun, 336000 Jiangxi China; 3 Engineering Technology Research Center of Jiangxi Universities and Colleges for Selenium Agriculture, Yichun, 336000 Jiangxi China

**Keywords:** Goat, Pathological injury, Heat shock proteins, Stomach, Transport stress

## Abstract

**Background:**

Transportation is necessary to introduce new breeds of goats to the farm and move the adult meat goat from the farm to the slaughterhouse. However, these actions may give rise to transport stress. Heat shock proteins (HSPs) are playing some important regulate roles during transport stress. The aim of this study was to evaluate the effects of transport stress on the pathological injury and HSPs expression in the stomach of goats. A total of three batches of Ganxi goats from western Jiangxi province were enrolled in this study. For each batch, twelve healthy adult male goats were randomly divided into three groups (four goats per batch and per group): Control group, stress group transported during 2 h and stress group transported during 6 h.

**Results:**

Our results showed that the different degrees of stomach walls damage, with the change of expression levels of heat shock protein 27 (HSP27), heat shock protein 70 (HSP70) and heat shock protein 90 (HSP90), occurred after goats transportation. In rumen, the mRNA and protein expressions of HSP27 and HSP70 were increased after transport stress, but not HSP90. In reticulum, all three HSPs mRNA and protein levels were upregulated after 2 h transport, but decreased after 6 h transport. In omasum, HSP27 and HSP70 mRNA and protein were increased after transport stress, however, HSP90 mRNA level only had a slightly enhancement after transport stress. In abomasum, HSP70 and HSP90 mRNA and protein levels were increased after transport stress, but HSP27 was decreased after transport stress.

**Conclusions:**

Taken together, these results revealed that the pathological changes in the gastric tissues and the stomach HSPs expression in goats are related to transport stress and duration. Moreover, this study also provides some new data to advocate reducing transport stress of goats and improving animal welfare.

## Background

As a part of the digestive system, the stomach plays an important role in the daily life of livestock. Ruminants that digest plant-based foods have a four-compartment stomach consisting of the rumen, reticulum, omasum, and abomasum [1]. Each compartment performs its own function via different component (such as microorganisms). Furthermore, the stomachs of ruminants are very sensitive to different stresses [2].

Transport, which is one of the stressors of livestock animals, can bring certain economic loss to animal husbandry [3]. During transportation, animals may expose to some stimuli such as changes in temperature and humidity, restriction of feed and water, mixed with other animals, and so on. These stimuli are highly stressful which can induce so called “transport stress” in animals and result in the increase of morbidity and even cause death [4, 5]. Above these stimuli will leads to not only physiological changes including blood composition, meat and skin quality, hormones, metabolites, enzymes, and live weight [6, 7], but also tissue and organ damages such as liver and jejunum [8, 9]. In addition, transport stress also can improve the level of reactive oxygen species (ROS), which can trigger the imbalance between oxidation and antioxidant defense systems, and eventually induce oxidative stress [10, 11]. ROS can cause DNA damage, lipid peroxidation and protein damage [12]. Oxidative stress caused by transportation has been reported to impair swine intestinal epithelial cells [9]. Moreover, previous studies have shown that the expressions of HSP27, HSP70 and HSP90 are significantly changed in pigs’ hearts or chickens’ brains during transport stress [13, 14]. Therefore, it is reasonable to presume that the expressions of HSP27, HSP70 and HSP90 may also change during transport stress in goats, playing some essential protective roles.

A better understanding of the effects of transport stress on pathological injury and expression of the main heat shock proteins in the caprine stomach would be of meaningful value to researchers to provide the basis for the design of novel strategies to minimize the adverse influence of transport stress on animals and improve the animal welfare. However, in our knowledge, they have not been reported yet. Therefore, this study aims to assess the pathological injury and the changes in HSP27, HSP70 and HSP90 expression in the caprine stomach after transport stress with different duration.

## Results

### Effect of transport stress on rumen pathological injury and HSPs expression

**Fig. 1:**
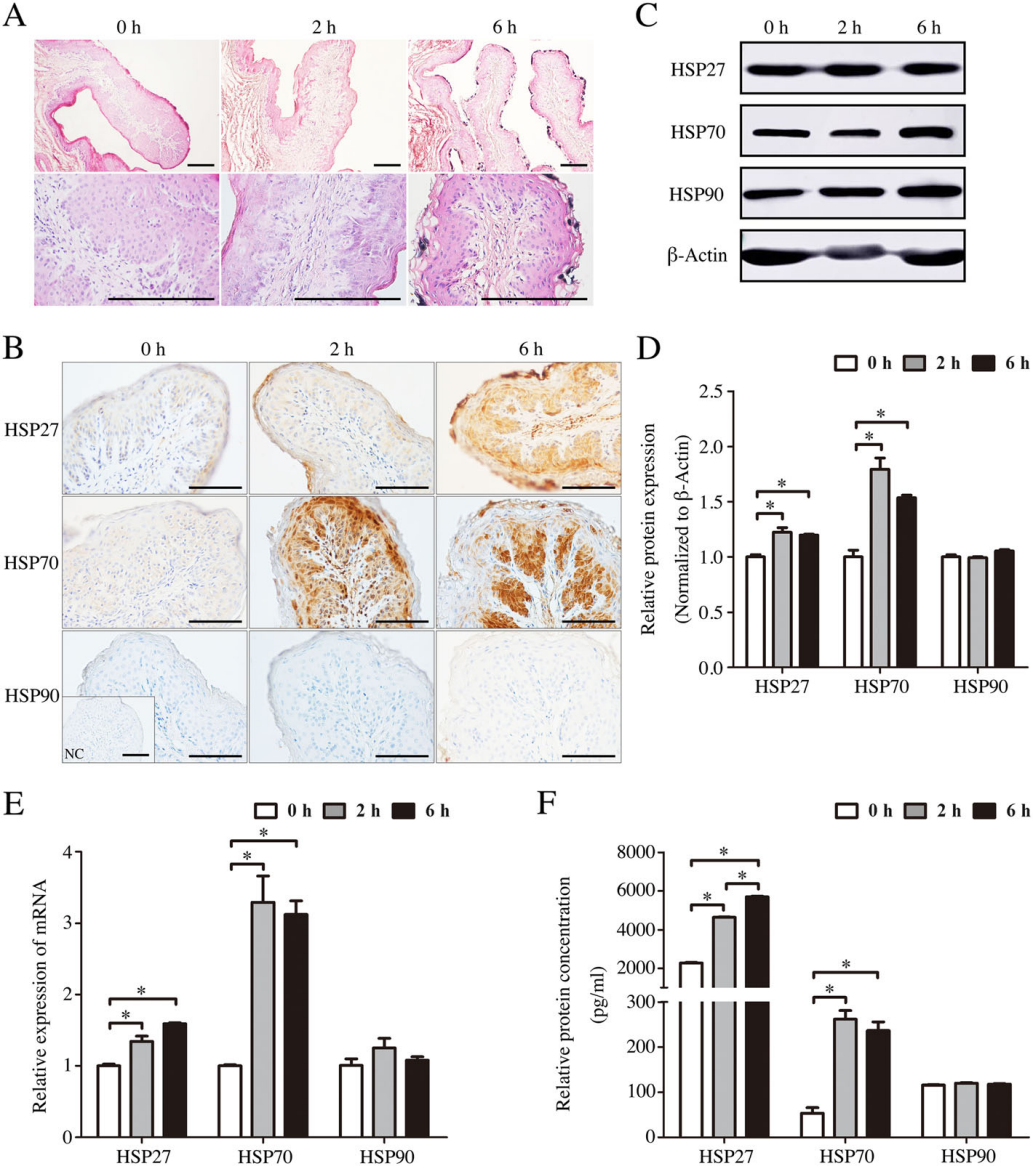
The effects of transportation-induced transport stress on rumen of goats. **a** Hematoxylin and eosin stained-rumen sections from control and transportation-induced transport stress goats. Scale bar 100 μm. **b** Immunohistochemical staining showing the expression of HSP27, HSP70 and HSP90 in rumen of goats from control group and transported groups. NC, negative control. Scale bar 50 μm. **c** Western blot of the HSP27, HSP70 and HSP90 proteins in the rumen of 2 h and 6 h transportation-treated goats. β-Actin was used as loading control. **d** Quantitative analysis of western blot using Image Pro Plus 6.0. **e** Real-time PCR analysis of the HSP27, HSP70 and HSP90 mRNA levels in the rumen of 2 h and 6 h transportation-treated goats. **f** ELISA analysis of the HSP27, HSP70 and HSP90 concentrations in the rumen of 2 h and 6 h transportation-treated goats. Data are presented as the means ± SD; *p < 0.05

Compared with control group, the rumen pathological injury of transported groups were significantly impaired. H.E. staining results indicated that the width of rumen papilla top of transported groups were decreased and tended to decrease papilla basal width compared with control group. The junction of the lamina propria cells was loose, and some blood cells were infiltrated into the lamina propria (Fig. 1a).

To evaluate the response of HSPs when animals are exposed to harmful stimulus, we detected the expression of HSPs protein and mRNA in rumen. There was a significant increase in HSP27 in the stratified squamous epithelium of rumen papilla after 6 h transportation. Moreover, the expression of HSP70 in the stratified squamous epithelium was also significantly elevated, and HSP90 had no significant change (Fig. 1b). Western blot analyses also showed that a significant increase in HSP27 and HSP70 (*p* < 0.05) in the rumen were induced after 2 h or 6 h transportation, but not HSP90 (*p* > 0.05; Fig. 1c, d). As expected, real-time PCR analyses showed that the levels of mRNA of HSP27 and HSP70 were highly increased in 2 h or 6 h transportation group (*p* < 0.05), but not HSP90 (*p* > 0.05; Fig. 1e). Finally, enzyme-linked immunosorbent assay (ELISA) assays also performed that obvious increases of HSP27 and HSP70 concentration (*p* < 0.05), but not HSP90 (*p* > 0.05), were observed in the rumen of 2 h and 6 h transportation-treated goats (Fig. 1f).

### Effect of transport stress on reticulum pathological injury and HSPs expression

**Fig. 2:**
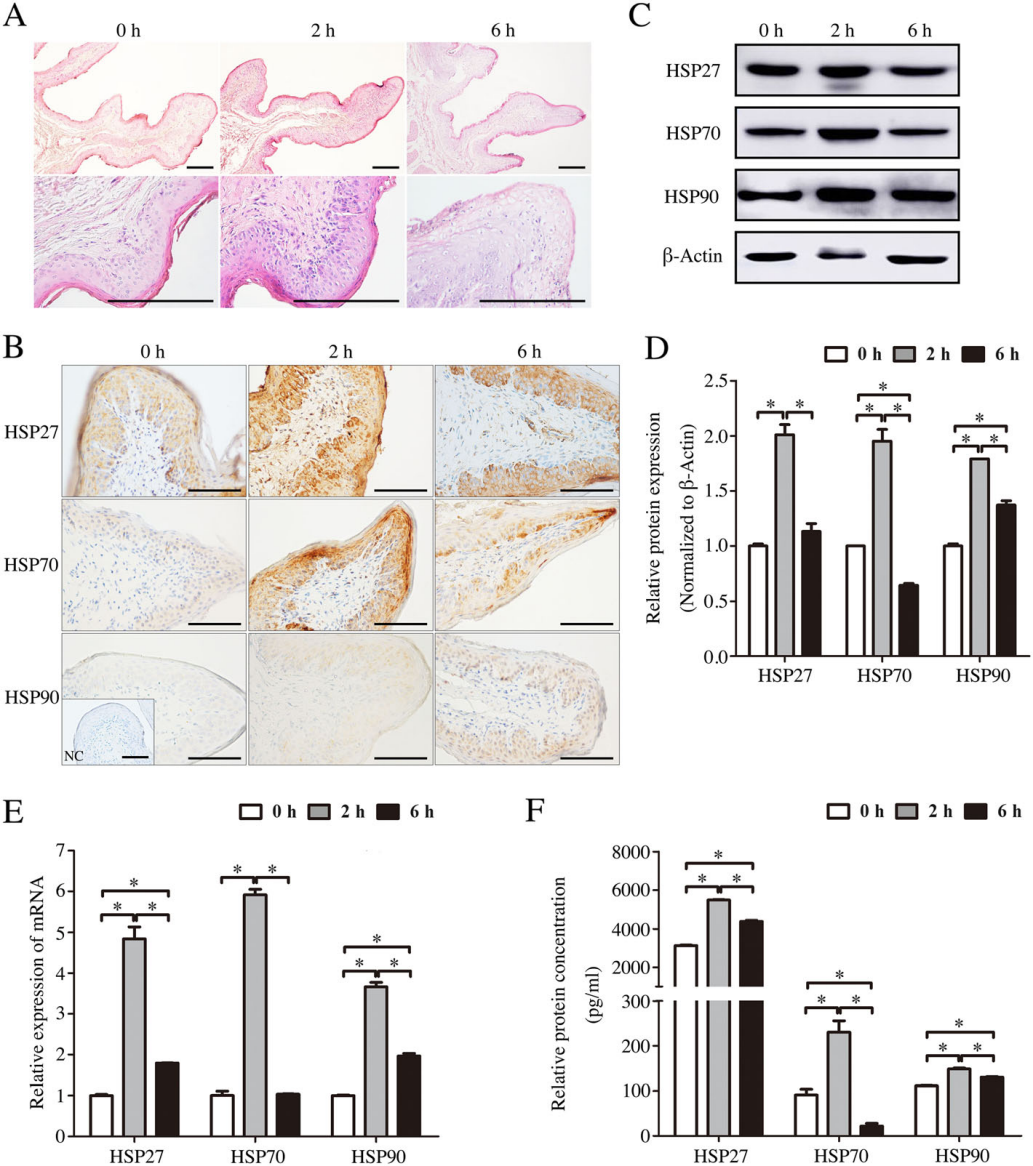
The effects of transportation-induced transport stress on reticulum of goats. **a** Hematoxylin and eosin stained-reticulum sections from control and transportation-induced transport stress goats. Scale bar 100 μm. **b** Immunohistochemical staining showing the expression of HSP27, HSP70 and HSP90 in reticulum of goats from control group and transported groups. NC, negative control. Scale bar 50 μm. **c** Western blot of the HSP27, HSP70 and HSP90 proteins in the reticulum of 2 h and 6 h transportation-treated goats. β-Actin was used as loading control. **d** Quantitative analysis of western blot using Image Pro Plus 6.0. **e** Real-time PCR analysis of the HSP27, HSP70 and HSP90 mRNA levels in the reticulum of 2 h and 6 h transportation-treated goats. **f** ELISA analysis of the HSP27, HSP70 and HSP90 concentrations in the reticulum of 2 h and 6 h transportation-treated goats. Data are presented as the means ± SD; *p < 0.05

H.E. staining results showed that the muscle bands were degenerated heavily after transportation compared with the control group. The stratified squamous epithelial cells of reticulum plica were degenerated and the karyolysis and vacuolation of cytoplasm were also observed in the process of transported stress, especially stress group transported during 6 h (Fig. 2a).

HSP27, HSP70 and HSP90 were mainly localized on the stratified squamous epithelium of plica (Fig. 2b). Western blot results indicated that the expression of HSP27, HSP70 and HSP90 proteins were increased after 2 h transportation (*p* < 0.05). In particular, HSP27 and HSP70 showed the highest expression in the stress group transported during 2 h compared with control group and stress group transported during 6 h. With the extension of transportation time, the protein levels of HSP27, HSP70 and HSP90 were markedly decreased in the stress group transported during 6 h when compared with stress group transported during 2 h (*p* < 0.05), even down to the initial levels (Fig. 2c, d). The changes of HSP27, HSP70 and HSP90 mRNA levels shared the same trend (Fig. 2e). ELISA analysis further confirmed the above results (Fig. 2f).

### Effect of transport stress on omasum pathological injury and HSP expression

**Fig. 3:**
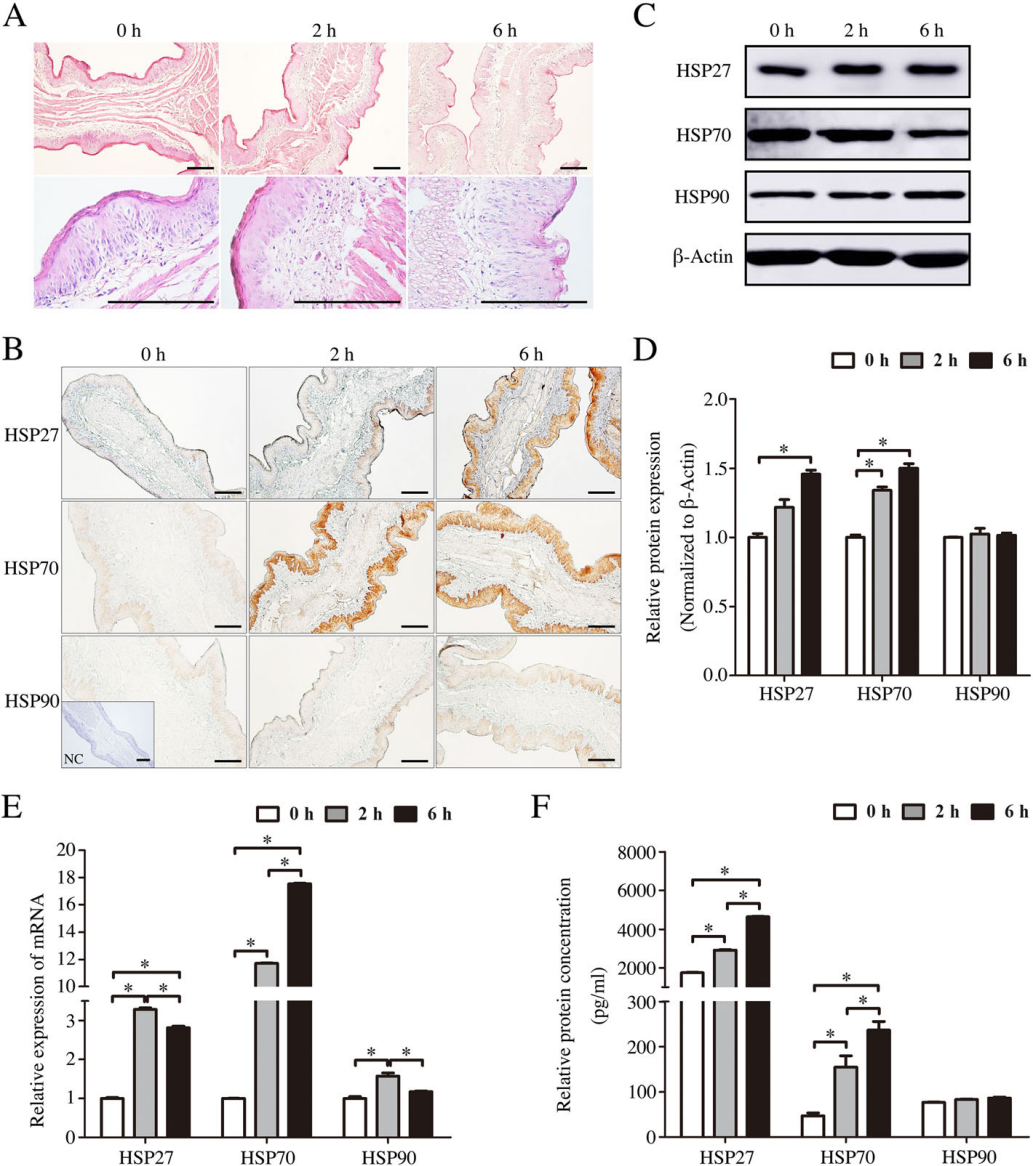
The effects of transportation-induced transport stress on omasum of goats. **a** Hematoxylin and eosin stained-omasum sections from control and transportation-induced transport stress goats. Scale bar 100 μm. **b** Immunohistochemical staining showing the expression of HSP27, HSP70 and HSP90 in omasum of goats from control group and transported groups. NC, negative control. Scale bar 50 μm. **c** Western blot of the HSP27, HSP70 and HSP90 proteins in the omasum of 2 h and 6 h transportation-treated goats. β-Actin was used as loading control. **d** Quantitative analysis of western blot using Image Pro Plus 6.0. **e** Real-time PCR analysis of the HSP27, HSP70 and HSP90 mRNA levels in the omasum of 2 h and 6 h transportation-treated goats. **f** ELISA analysis of the HSP27, HSP70 and HSP90 concentrations in the omasum of 2 h and 6 h transportation-treated goats. Data are presented the means ± SD; *p < 0.05

Compared with the control group, the cristae of the omasum leaflet was degenerated after transportation. The vacuolation was obviously observed in the muscular layer of the leaflet of stress group transported during 6 h. The karyolysis and vacuolation were also observed in the epithelial cells cytoplasm, and most of blood cells were infiltrated into the lamina propria (Fig. 3a).

Immunostaining analysis indicated that three HSPs were located in the epithelium of the leaflet. The expressions of HSP27 and HSP70 were elevated after transport treatment, but the expression of HSP90 was not significantly changed (Fig. 3b). These results were further confirmed by Western blot at the protein expression level (Fig. 3c, d). Real-time PCR results performed the same variation trend of these molecules mRNA and protein levels (*p* < 0.05; Fig. 3e). ELISA also further confirmed the above results (Fig. 3f).

### Effect of transport stress on abomasum pathological injury and HSP expression

**Fig. 4:**
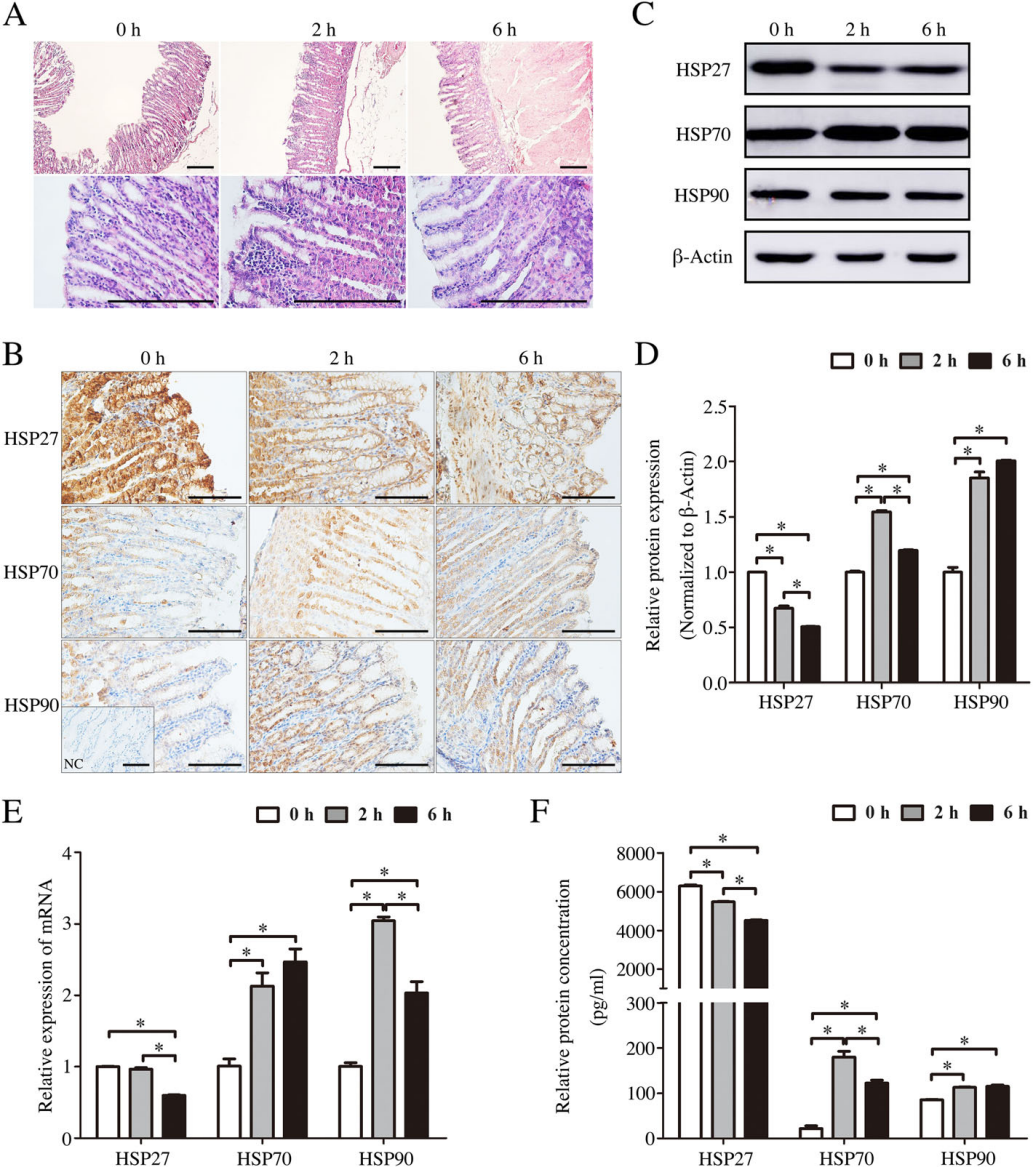
The effects of transportation-induced transport stress on abomasum of goats. **a** Hematoxylin and eosin stained-abomasum sections from control and transportation-induced transport stress goats. Scale bar 100 μm. **b** Immunohistochemical staining showing the expression of HSP27, HSP70 and HSP90 in abomasum of goats from control group and transported groups. NC, negative control. Scale bar 50 μm. **c** Western blot of the HSP27, HSP70 and HSP90 proteins in the abomasum of 2 h and 6 h transportation-treated goats. β-Actin was used as loading control. **d** Quantitative analysis of western blot using Image Pro Plus 6.0. **e** Real-time PCR analysis of the HSP27, HSP70 and HSP90 mRNA levels in the abomasum of 2 h and 6 h transportation-treated goats. **f** ELISA analysis of the HSP27, HSP70 and HSP90 concentrations in the abomasum of 2 h and 6 h transportation-treated goats. Data are presented as the means ± SD; *p < 0.05

Compared with the control group, the abomasum morphology in the transported groups were significantly impaired after transport under the microscope. The width of lamina propria and gastric glands were significantly decreased along with the infiltration of inflammatory cells to lamina propria. The exfoliation of chief cells can be found in the end of fundic gland, and some epithelial cells were degenerated and the karyolysis and vacuolation of cytoplasm were also observed (Fig. 4a).

Immunostaining results indicated that the cytoplasm localization of HSP27 in the gastric glandular cells was a significantly decreased in the transportation-treated group, but HSP70 and HSP90 were increased (Fig. 4b). Western blot results were further supported this conclusion. However, compared with stress group transported during 2 h, the protein level of HSP27 of stress group transported during 6 h was decreased (*p* < 0.05; Fig. 4c, d). Real-time PCR results also showed that the mRNA level of HSP27 was decreased after 6 h transported (*p* < 0.05). On the contrary, HSP70 and HSP90 mRNA levels were increased after transport stress (*p* < 0.05; Fig. 4e). ELISA results also performed the same variation trend of these HSPs protein levels, consisting with western blot results (Fig. 4f).

## Discussion

In this study, our results show that the morphological damage and the changes of some important HSP expression in goat stomach can be caused by transport stress. Some studies have demonstrated that transportation is a strong stress due to exposure of the animals to unfavorable environments [15]. However, the concrete damage of four stomachs of goats during transportation was less reported. This study for the first time showed the effect of transport stress on the morphological damage and the expression changes of HSP27, HSP70 and HSP90 in goat’s stomach.

The function of the goat stomach has been studied, but the specific function of the stomach has not been fully understand so far [16, 17]. Previous study has demonstrated that the activity of the hypothalamic-pituitary-adrenal axis as well as changes in gastrointestinal tissue can be increased by physiological responses to stressors. And an increase in adrenocortical activity is related to an increase in the incidence of gastric ulceration [18]. Otherwise, an interesting research performed that the biomechanical properties of the gastric strips can be changed by stress in the stomach of rat and rabbit [19], which will affect the animals’ ability to gather and digest food. The rumen is a complex anaerobic microbial ecosystem in ruminants. Rumen as one of the fore-stomach of ruminants was the first stomach, which played some important roles in fermentation and fatty acid (FA) synthesis [20]. The main functional part of rumen is the rumen papilla, which is consists of two compartments named stratified squamous epithelium and lamina propria [1, 21]. Previous study has performed that the width of papilla can be decreased by heat stress in growing Holstein calves [22]. In our study, the width of papilla top was also decreased by transport stress. But in lambs, the papilla width was not affected by mild heat stress [23]. Otherwise, stratified squamous epithelium and lamina propria were also impaired. Another fore-stomach of ruminants was reticulum, which is located at the front of the rumen and pathological injury was also induced by transport stress. The tissue structure of reticulum gastric mucosa is basically similar with rumen. The muscle bands are located in the plica top and the cristae protrude from both sides of the plica [24]. As expected, the width of plica top was decreased, and the muscle bands were also impaired after transportation. As the smallest compartment of the forestomach, omasum has some important roles such as the absorption of water and electrolytes [25, 26]. Our data indicated that the leaflet of omasum was damaged after transportation. The muscle layer was highly vacuolated after 6 h of transportation. The rate of the apoptosis in the epithelium was significantly increased. Abomasum is the last stomach of ruminants, which is mainly responsible for the absorption of some nutrients. The structure of ruminant abomasum is the same with the stomach of monogastric animal, which mainly contains gastric pit, gastric gland (fundic gland, cardiac gland and pyloric gland) and lamina propria. A previous study has demonstrated that acute exudation can be observed in the mucous lamina propria and submucosa of stomach of transported piglets after 2 h transportation [15]. Exfoliation of chief cells also can be found in the gastric surface of pigs after transportation [27]. In our study, gastric glands were also impaired, and the exfoliation of chief cells also can be found at the bottom of fundic gland. Therefore, we speculated that the issues of livestock caused by transportation may be mainly related to the damage of stomach. To our knowledge, this is the first controlled experiment in which a response of caprine stomach pathological injury to transport stress has been evaluated.

The HSP/chaperone network is a major component of multiple stress responses [28]. Transport stress response is characterized by a series of factors such as vertigo and high temperature, which also involves the change of heat shock proteins [29, 30]. In earlier studies, HSP27 is considered as an ATP-independent molecular chaperone which was involved in the protein refolding machinery [31, 32]. HSP27, a well-known antiapoptotic and antioxidant protein, is deeply involved in the regulation of cytoskeletal organization [33–35]. The expression of HSP27 in rat intestine was highly increase after transportation [30]. In our study, the expression of HSP27 was also increased in the goat rumen, reticulum and omasum. The expression of HSP27 is decreased in the pig heart after transportation [13]. We also found that the expression of HSP27 in the abomasum was decreased after transportation. These results suggest that the increase of HSP27 is able to protect the rumen, the reticulum and the omasum from damages of the transport stress. The decrease of HSP27 in the abomasum indicates that the transport stress may have overcharged the repair mechanisms of the cells. HSP70s play key roles in cellular development and protect living organisms from environmental stresses. In pigs, the expression level of HSP70 in blood lymphocytes is significantly increased under cold stress [36]. The protein level of HSP70 is also increased in the rat intestine and the goat kidney after transportation [30, 37]. In our study, the increase of HSP70 expression the goat stomachs may be able to makes a protective response to the transport stress. HSP90 is a molecular chaperone that is involved in the activation of disparate client proteins distributed in stressed cells [38–40]. The level of HSP90 is also increased in the rat small intestine after transportation [30]. In our study, we found that the mRNA and protein levels of HSP90 were not significantly changed in the rumen and the omasum after transportation. Although HSP90 was increased in the reticulum and the abomasum, but the level was still very low.

## Conclusion

In summary, our data indicated that goat transportation-induced transport stress may lead to some adverse effects such as the pathological changes in the gastric tissue followed by the change of stomach HSP27, HSP70 and HSP90 expression in goats. Furthermore, heat shock proteins can play some crucial roles in protecting stomachs from damage caused by the transport stress. Further research will focus on the exploration of specific protective mechanism.

## Methods

### Animals and experimental design

According to previous literature [9, 13], to make the study statistically significant, a total of three batches of Ganxi goats were enrolled in this study. For each batch, twelve healthy adult male goats were obtained from the same farm (Mulei) of western Jiangxi province, which were of similar body weight (13.89 ± 2.96 kg) and age (1-year-old). Twelve goats per batch were randomly enrolled into three groups: the control group (*n* = 4), the stress group transported during 2 h (*n =* 4), and the stress group transported during 6 h (*n =* 4). Transport treatment was performed as previously described with some modifications [15]. Briefly, the control group were directly transported to the abattoir (1.5 h journey) a day prior to slaughter and housed in resting pens at room temperature for 24 h (16:00 to next day 16:00) with water ad libitum before slaughter. The transported groups were transported on the road at 28–32 °C with the speed of 35–45 km/h and there were no food and water for the goats during transportation. No accidents occurred during the transport period. After transportation, both stress groups had no rest and all of the three groups’ goats were euthanized while under anesthesia by i.v. injection with pentobarbital sodium at 90 mg/kg. Death was confirmed by auscultation for cardiac arrest. The method of euthanasia is consistent with the recommendations by the Chinese Association for Laboratory Animal Sciences.

### Samples collection

The rumen, reticulum, omasum and abomasum samples were collected by sterile surgical instruments. Then all of these tissues were flushed with a physiological saline to remove remains of contamination. The tissue collection method was performed as previously described [41]. Tissue slices of two centimeters in length were prepared and subsequently fixed in 4% paraformaldehyde for H.E. staining and immunohistochemistry analyses. Tissue slices of one centimeters in length were prepared and subsequently frozen in liquid nitrogen for HSPs protein and mRNA expression analyses.

### Paraffin sections preparation

The paraffin sections preparation was performed as previously described [42]. The tissue samples fixed in 4% paraformaldehyde were rinsed with running water, followed by dehydration in different concentrations of alcohol and soaking in xylene and paraffin, tissues were embedded in liquid paraffin. Following solidification, paraffin-embedded tissue samples were cut into a series of 5 μm sections using a rotary microtome (RM2235, Leica Biosystems, Buffalo Grove, IL, USA). Finally, all the paraffin sections were stored at 4 °C for further research.

### Hematoxylin-eosin staining (H.E. staining)

The H.E. staining was performed as previously described [42]. The paraffin sections (5 μm) were deparaffinized in xylene and rehydrated with a graded series of ethanol, washed with water, and then stained with hematoxylin for 10 min. Next, the sections were washed with water and immersed in a hydrochloric acid-alcohol solution. After washing with water, the sections were stained with eosin for 40 s. Subsequently, the sections were decolorized in water for 3 min and dehydrated gradually in a series of different concentrations of alcohol. Finally, the sections were soaked in xylene until transparent and sealed with neutral resin. The sections were viewed and photographed using a digital optical microscope system. Then picked out some representative images and put in the text. The degree of alteration was evaluated through H.E. staining of all tissue specimens.

### Immunohistochemistry

Immunohistochemistry was performed as previously described [43, 44]. The paraffin sections (5 μm) were deparaffinized in xylene, rehydrated with a graded series of ethanol, and washed in water. Antigen retrieval was performed by microwaving the sections in 10 mM sodium citrate buffer (pH 6.0) followed by cooling the sections to room temperature. The endogenous horse radish peroxidase activity was inhibited with 3% H_2_O_2_ for 15 min. After nonspecific binding was blocked with 10% horse serum at 37 °C for 1 h, the sections were incubated with primary antibody at 4 °C overnight. The primary antibodies used in this study include mouse anti-HSP27 antibody (1:800; ab79868, Abcam, Cambridge, UK), mouse anti-HSP70 antibody (1:800; ab5439, Abcam) and mouse anti-HSP90 antibody (1:400; ab13492, Abcam). The sections were then incubated with a biotin-labeled goat anti-mouse IgG antibody (1:1000; ab6789, Abcam) and a streptavidin-conjugated HRP complex (SP9002, Zhongshan Golden Bridge, Beijing, P. R. China). Finally, the signals were visualized with the DAB Horseradish Peroxidase Color Development Kit (ZLI9018, Zhongshan Golden Bridge) and counterstained with hematoxylin.

### Total RNA extraction and cDNA synthesis

The total RNA was isolated using the HP Total RNA Kit (R6812, Omega, Guangzhou, China). RNA concentration was determined using the spectrophotometer (NanoDrop 2000, NanoDrop Technologies Inc., Wilmington, DE, USA) with 260/280 ratios of 1.8–2.0 considered acceptable. Next, 3.0 μL DNase 10× Reaction Buffer, 3.0 μL RNase-free DNase (M6101, Promega, Beijing, China), 0.5 μL Recombinant Rnase Inhibitor (2313A, TaKaRa, Dalian, China) and 0.5 μL 50 mM DL-Dithiothreitol (D9779, Sigma, St. Louis, Missouri, USA) were used to remove the chromosomal DNA in 5 μg of RNA samples (37 °C, 30 min). Thereafter, RNA samples were reverse-transcribed into cDNA with the *TransStart*® First-Strand cDNA Synthesis SuperMix (AT301, TransGen, Beijing, China), and stored at − 80 °C for real-time polymerase chain reaction (RT-PCR) analysis.

### RT-PCR analysis

For RT-PCR, primer sequences used were listed in Table 1. The cDNA was amplified using a *TransStart*® Top Green qPCR SuperMix (AQ131, TransGen) on the CFX96 Touch™ Real-Time System (Bio-Rad, Richmond, CA, USA). The RT-PCR parameters were as follows: 95 °C for 3 min, then 40 cycles of 95 °C for 10 s (denaturation) and 60 °C for 30 s (annealing). Melting curve analysis comprised of the following parameters: 95 °C for 10 s, after which the ramp speed was decreased from 1.667 °C/sec to 0.01667 °C/sec and data were collected continuously until it reached 95 °C where the temperature was held for 30 s, and finally held at 60 °C for 15 s. The data from RT-PCR were analyzed using the 2^-ΔΔCt^ method. The relative expression levels were normalized to the expression level of β-Actin.

**Table 1:** The primer sequence of the target gene

Target Genes ^**a**^	Primer sequence (5′-3′)	Accession No.^**b**^	Product Length, bp
*HSP27*	Forward (F): CCTGGACGTCAACCACTTC Reverse (R): GCTTGCCAGTGATCTCCAC	JQ957566.1	76
*HSP70*	F: ACGTTCGACGTGTCCATTCT R: TCACCAGCCTGTTGTCGAAG	NM_001285703.1	106
*HSP90*	F: CAAGAGCCTGACCAACGACT R: AAAGGAGCTCGTCTTGGGAC	AF548366.1	108
*β-Actin*	F: CTCTTCCAGCCTTCCTTCCT R: GGGCAGTGATCTCTTTCTGC	NM_001314342.1	177

^a^HSP27, 70, 90 = heat shock protein 27, 70, 90; ^b^ the reference sequence number is listed for primers whose source is the National Center for Biotechnology Information (NCBI) GenBank database (http://www.ncbi.nlm.nih.gov/genbank/)

### Protein extraction and western blot analysis

Western blot was performed as previously described [45, 46]. Briefly, the tissues were homogenized in ice-cold homogenization lysis buffer (150 mM NaCl; 50 mM Tris-HCl, pH 7.5; 1% Triton X-100; and 0.25% sodium deoxycholate), which contained the Protease Inhibitor Cocktail (Roche, Penzberg, Germany). After putting on ice for 30 min, the tissue lysates were centrifuged (12,000×g) at 4 °C for 15 min, and the supernatant was obtained. The protein concentrations were measured by the BCA Kit (P1511, Applygen Technologies Inc., Beijing, China). The protein samples were mixed with 5× loading buffer and denatured at 100 °C for 15 min. Then the protein samples were separated on 10% sodium dodecyl sulfate-polyacrylamide gel electrophoresis (SDS-PAGE) gels at 100 V for 30 min, then 120 V for 1.5 h. Subsequently, the separated proteins were transferred onto polyvinylidene difluoride (PVDF) membranes (Millipore Sigma, Bedford, MA) with 0.45 μm apertures at 200 mA for 1.5 h at 4 °C. After transfer, 5% non-fat dried milk and 0.1% Tris-Buffered-Saline with Tween (TBST) were used to block the membrane at room temperature for 2 h. The membranes were washed with TBST and then incubated with primary antibody overnight at 4 °C. The primary antibodies used in this study include mouse anti-HSP27 antibody (1:2000; ab79868, Abcam), mouse anti-HSP70 antibody (1:2000; ab5439, Abcam), mouse anti-HSP90 antibody (1:1000; ab13492, Abcam) and mouse anti-β-Actin antibody (1:1000; BM0627, Boster, Wuhan, China). After the membranes were incubated with an HRP-conjugated goat anti-mouse IgG (secondary antibody, 1:5000; BA1051, Boster) at room temperature for 2 h, the blot was washed with wash buffer and detected by enhanced chemiluminescence (ECL) using Immobilon Classico Western HRP Substrate (WBLUC0500, Millipore, Danvers, MA, USA). An imaging system (Amersham Imager 600, General Electric, Boston, Massachusetts, USA) was used for the record of the ECL signals and the Image Pro Plus 6.0 software (Media Cybernetics, USA) was utilized for analysis. The protein levels were expressed as a fold change compared with the average value of the control group (0 h).

### HSPs assay

The concentrations of HSP27, HSP70 and HSP90 in tissues were assayed by a commercial Goat HSP27, HSP70 and HSP90 ELISA Kits (Ai Pu Rui Sheng Biotech Co., Ltd., Beijing, China). Carry out the specific operation according to the manufacturer’s instructions.

### Statistical analysis

All the data of this study were presented as the means ±standard deviation (SD) and analyzed using GraphPad Prism 5.01 software (GraphPad Software Inc., San Diego, CA, USA). The SPSS 18.0 software (SPSS Inc., Chicago, IL, USA) was used for data analysis, single factor variance analysis after LSD multiple comparisons. The data were statistically analyzed by two-way ANOVA. A *p* value < 0.05 was considered statistically significant.

## Supplementary information

**Additional file 1.** Western blot instructions and original images.

## Data Availability

The nucleotide sequences used in this study were collected from the National Center for Biotechnology Information (NCBI) GenBank repository. The GenBank accession numbers of all sequences were listed in the Table 1. The commercial antibodies used in this study were listed in the “Protein extraction and western blot analysis” section. The datasets used and/or analyzed during the current study are available from the corresponding author on reasonable request.

## Abbreviations

HSP: Heat shock protein
ROS: Reactive oxygen species
FA: Fatty acid
