# Supplementary material for: Effects of transport stress on pathological injury and expression of main heat shock proteins in the caprine stomach
Source: BMC Vet Res. 2020 Sep 22;16:347. doi: 10.1186/s12917-020-02569-z (PMC7507251; doi:10.1186/s12917-020-02569-z)

**Western Blot Instructions and Original Images**

At first, we check the full films of western blot for the protein samples of goats to ensure the specificity of antibodies. The results were showed as follows:


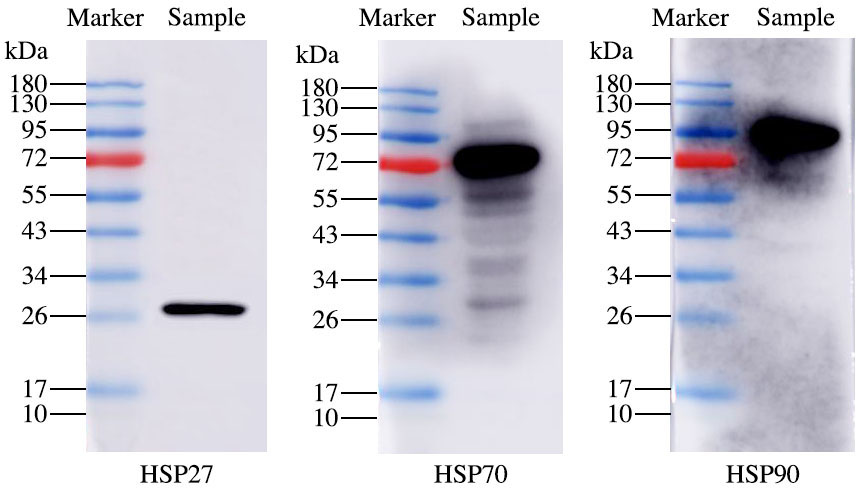


After ensuring the specificity of the anti-HSP27, anti-HSP70 and anti-HSP90, we performed following experiments in article with cropped gels. All original images were showed as follows:

1. **Rumen**


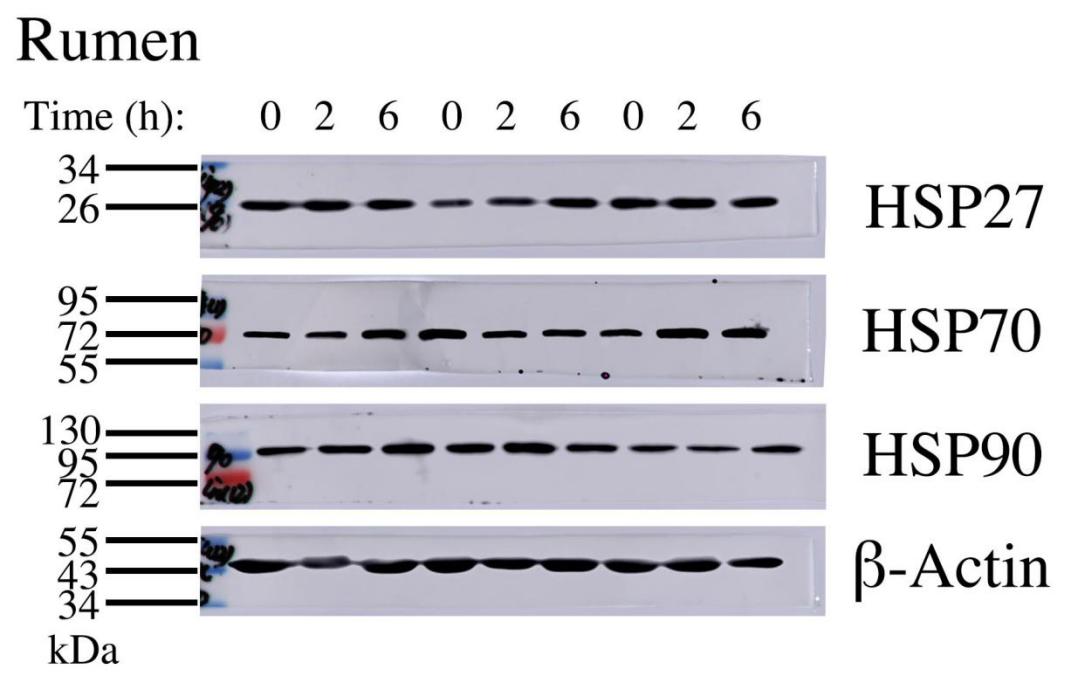


“Time” represents the time of transport. This figure was showed the result of nine goats from three groups. **This interpretation is also applied to the following figures.**

1. **Reticulum**


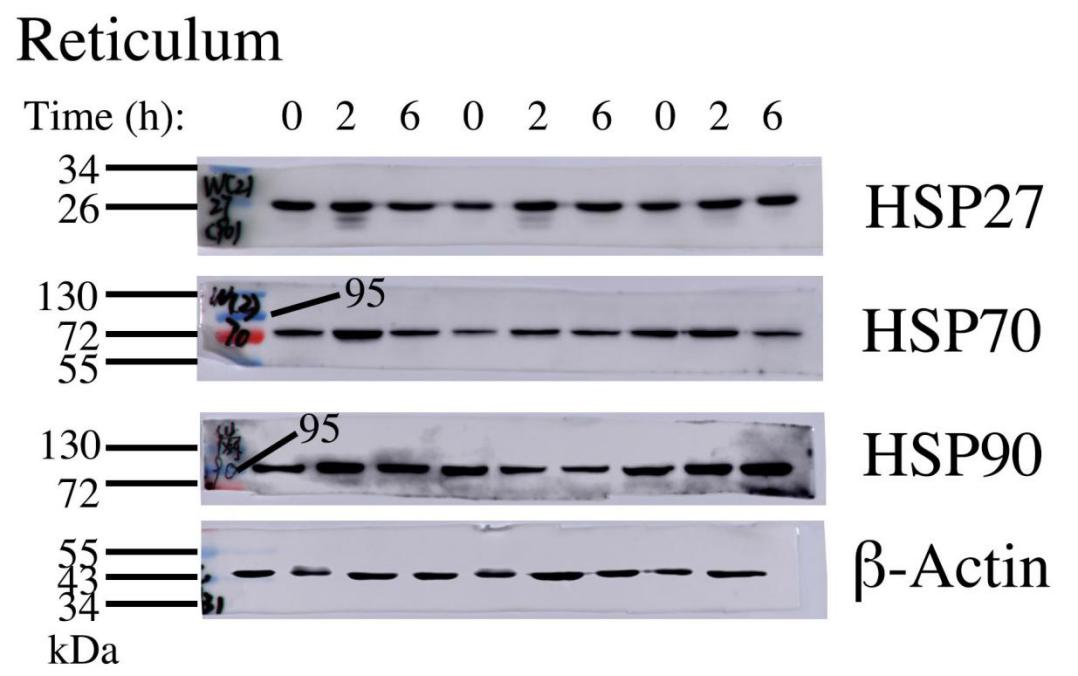


1. **Omasum**


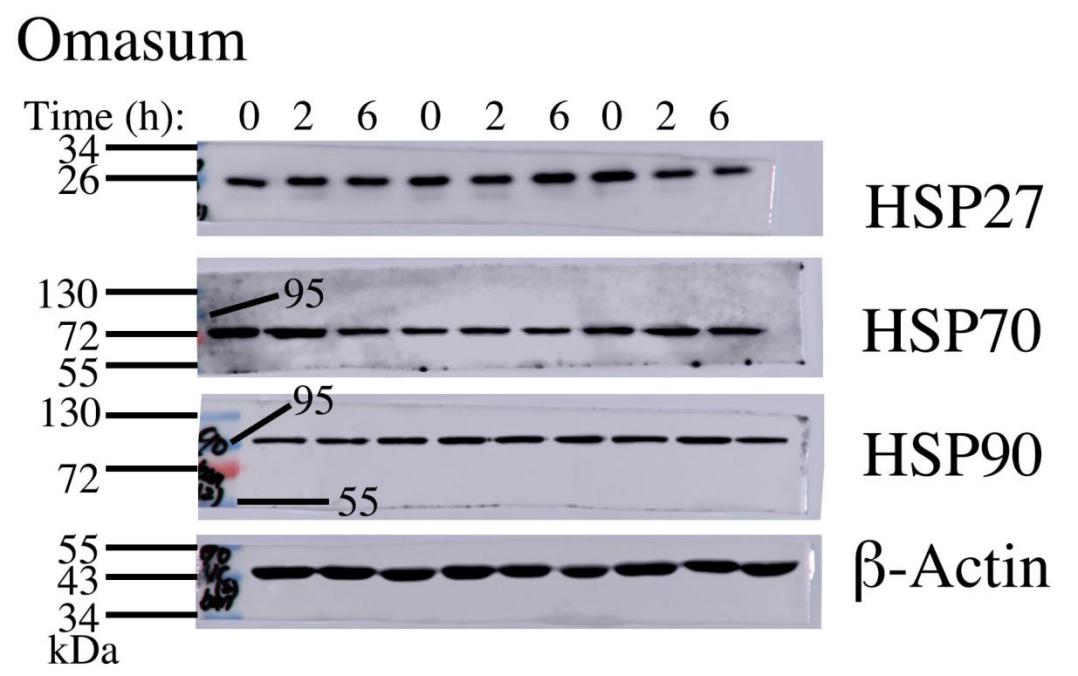


1. **Abomasum**


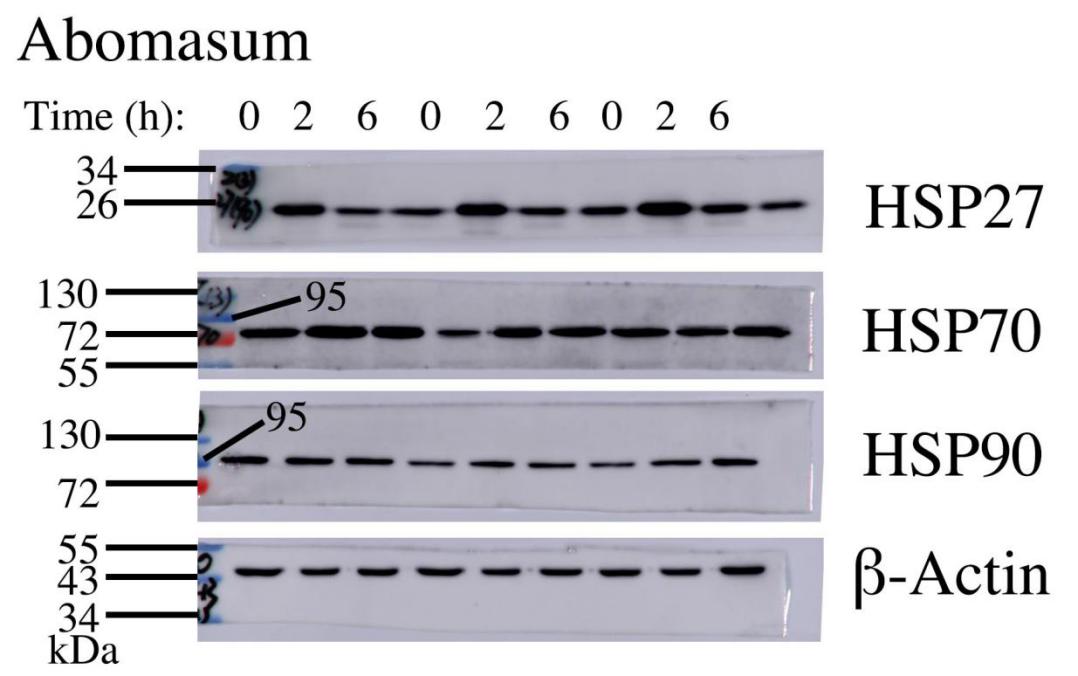

Supplement: Supplementary file 1 — Additional file 1. Western blot instructions and original images. [file 12917_2020_2569_MOESM1_ESM.docx]
